# TIGIT and PD-1 Immune Checkpoint Pathways Are Associated With Patient Outcome and Anti-Tumor Immunity in Glioblastoma

**DOI:** 10.3389/fimmu.2021.637146

**Published:** 2021-05-07

**Authors:** Itay Raphael, Rajeev Kumar, Lauren H. McCarl, Karsen Shoger, Lin Wang, Poorva Sandlesh, Chaim T. Sneiderman, Jordan Allen, Shuyan Zhai, Marissa Lynn Campagna, Alexandra Foster, Tullia C. Bruno, Sameer Agnihotri, Baoli Hu, Brandyn A. Castro, Frank S. Lieberman, Alberto Broniscer, Aaron A. Diaz, Nduka M. Amankulor, Dhivyaa Rajasundaram, Ian F. Pollack, Gary Kohanbash

**Affiliations:** ^1^ Department of Neurological Surgery, University of Pittsburgh, Pittsburgh, PA, United States; ^2^ Departments of Neurological Surgery, University of California, San Francisco, CA, United States; ^3^ University of Pittsburgh Medical Center (UPMC) Hillman Cancer Center Biostatistics Facility, University of Pittsburgh, Pittsburgh, PA, United States; ^4^ Department of Pediatrics, University of Pittsburgh, Pittsburgh, PA, United States; ^5^ Departments of Neurology, University of Chicago, Chicago, IL, United States; ^6^ Department of Neurology, University of Pittsburgh, Pittsburgh, PA, United States; ^7^ Department of Pediatrics, Division of Health Informatics, Children’s Hospital of Pittsburgh, University of Pittsburgh School of Medicine, Pittsburgh, PA, United States

**Keywords:** glioblastoma, immunotherapy, PD1, TIGIT, MDSCs, myeloid suppressor cell, gene network analyses

## Abstract

Glioblastoma (GBM) remains an aggressive brain tumor with a high rate of mortality. Immune checkpoint (IC) molecules are expressed on tumor infiltrating lymphocytes (TILs) and promote T cell exhaustion upon binding to IC ligands expressed by the tumor cells. Interfering with IC pathways with immunotherapy has promoted reactivation of anti-tumor immunity and led to success in several malignancies. However, IC inhibitors have achieved limited success in GBM patients, suggesting that other checkpoint molecules may be involved with suppressing TIL responses. Numerous IC pathways have been described, with current testing of inhibitors underway in multiple clinical trials. Identification of the most promising checkpoint pathways may be useful to guide the future trials for GBM. Here, we analyzed the The Cancer Genome Atlas (TCGA) transcriptomic database and identified PD1 and TIGIT as top putative targets for GBM immunotherapy. Additionally, dual blockade of PD1 and TIGIT improved survival and augmented CD8^+^ TIL accumulation and functions in a murine GBM model compared with either single agent alone. Furthermore, we demonstrated that this combination immunotherapy affected granulocytic/polymorphonuclear (PMN) myeloid derived suppressor cells (MDSCs) but not monocytic (Mo) MDSCs in in our murine gliomas. Importantly, we showed that suppressive myeloid cells express PD1, PD-L1, and TIGIT-ligands in human GBM tissue, and demonstrated that antigen specific T cell proliferation that is inhibited by immunosuppressive myeloid cells can be restored by TIGIT/PD1 blockade. Our data provide new insights into mechanisms of GBM αPD1/αTIGIT immunotherapy.

## Introduction

Malignant gliomas are the most common primary malignant central nervous system (CNS) tumor in adults (1). Glioblastoma (GBM) are highly aggressive brain cancers and the most common type of high-grade glioma (HGG) (2). The current standard of care for GBM patients include a combination of surgery, radiation therapy, and chemotherapy. However, even with standard of care, the median overall survival times remain less than two years (3, 4). Therefore, identification of novel GBM treatment strategies is warranted.

The immune system can mount specific and durable responses against tumors (5, 6). However, cancer cells, tumor-myeloid cells, and tumor infiltrating regulatory T cells (Tregs) can express negative regulators of the immune system including immune checkpoint (IC) molecules, thereby limiting effective anti-tumor immunity (7, 8). In recent years, the development of immunoregulatory drugs that block IC pathways, such as PD1/PD-L1 inhibitors, have emerged as a promising treatment strategy against a variety of malignancies, including melanoma, lung cancers, and head and neck cancers (9, 10). Although anti-PD1/PD-L1 immunotherapy shows durable response in other types of malignancies, its efficacy is limited to approximately 10% of GBM patients (11–13), thus highlighting the need for more effective and novel approaches, including the combination of additional IC inhibitors (ICIs) to target several IC pathways simultaneously.

T cell immunoreceptor with Ig and ITIM domain (TIGIT) is an IC receptor expressed on activated T cells, NK cells, and Tregs (14). Elevated TIGIT expression on TILs correlates with reduced TIL cytokine production and poor overall survival (14). TIGIT binds with high-affinity to CD155 (PVR) and with low-affinity to CD112 (PVRL2; nectin-2) which are expressed in the tumor microenvironment (TME) by antigen presenting cells (APCs) and tumor cells (15). The binding of TIGIT to CD155 suppresses the activation of TILs. CD155 can also bind CD226, which is expressed on T cells and provides a stimulatory signal which promotes T cell activation, thus competing with TIGIT binding to CD155. However, TIGIT has a significantly greater affinity to CD155 than CD226 (15, 16). While blocking the interaction between TIGIT and CD155 has been identified as a potential therapeutic target in treatment of malignancies, its effects in GBM are poorly understood (17).

MDSCs are myeloid-lineage regulatory cell that act as negative immune regulators in the TME (18). MDSCs consist of two major subtypes based on phenotype: PMN-MDSCs matched with granulocytes, and Mo-MDSC resembling inhibitory monocytes (18). In mice, PMN-MDSC are defined a CD11b^+^Ly6C^low^Ly6G^high^, and Mo-MDSC as CD11b^+^Ly6C^high^Ly6G^low^ whereas in human, PMN-MDSC are defined as CD14^-^CD11b^+^CD33^+^CD15^+^ and Mo-MDSC as CD14^+^CD11b^+^HLA-DR^low^. Some studies have shown that increased presence of MDSCs within the TME is related to poor clinical outcome in patients treated with ICI (19). Consequently, reduced infiltration of MDSCs in TME has shown enhanced anti-tumor efficacy of ICI in pre-clinical tumor models (20, 21).

In order to identify putative IC targets in GBM, we first analyzed of The Cancer Genome Atlas (TCGA) dataset and identified IC molecules whose expression is associated with poor survival in GBM patients. We found that upregulated expression of *PD1* and *TIGIT*, but not other ICs or their ligands, are associated with reduced patient survival. We demonstrate that dual treatment with αPD1/αTIGIT prolonged survival in a murine GBM model, at least in part by targeting MDSCs. Together, our data provide new insights into mechanisms of immunotherapy in GBM.

## Materials and Methods

### TCGA Data Analysis

The Cancer Genome Atlas (TCGA) database was used to assess survival of patients with GBM in accordance with gene expression levels of immune checkpoint molecules. Survival analysis was performed through the cBioPortal platform using a z-score of 1.0 for all checkpoint receptors and their respective ligands. The correlation of checkpoint gene expression with z score >2.0 was considered upregulated expression. Kaplan-Meier (KM) survival curves were generated to determine overall survival (OS) and disease-free survival (DFS).

### RNA Sequencing (RNA-seq) and Pathway Analysis

The study uses RNA-seq datasets of GBM tissue from The Cancer Genome Atlas (TCGA), and the raw expression files were downloaded from TCGA Genomic Data Commons (GDC) Data Portal. Reads were quantified and mapped to human genome (Ensembl GRCh38 Homo sapiens) Salmon version 0.8.2 (22). Transcript-per-kilobase-million (TPM) were used for gene-correlation and pathway analyses. Pearson’s rank correlation analysis was performed for *TIGIT* and *PDCD1*. Genes with statistically significant correlation (p value < 0.05 and false discovery rate (FDR) p < 0.05) were used to determine pathway enrichment using Gene Ontology (GO) (23) for Reactome (version 65 Released 2020-11-17) (24) and PANTHER Overrepresentation Test (Released 20200728) (25) curated pathways. Pathway enrichment cutoff was set for p<0.05 using Fisher’s Exact test and FDR p<0.05 and enrichment scores greater than 1. Immunological network analysis was performed using ClueGo v2.5.7 (26) and Cytoscape 3.8 (27) with the current parameters: GO ImmuneSystemProcess EBI-UniPort, GO term fusion, network specificity was set to medium-detailed, pathways’ p value <0.05 with Benjamini-Hochberg correction. Positively and negatively correlated genes were used to determine positively and negatively associated networks, respectively.

### Single Cell RNA-seq (scRNA-seq) Analysis

scRNA-seq data were obtained from Wang et al. (28) and processed as described previously (28). Briefly, the neoplastic cells and non-neoplastic cells were separated *via* copy number variation (CNV). The presence/absence of CNVs was assessed with CONICSmat (29), and the primary cell types of non-neoplastic cells (i.e. monocytes/myeloid) were identified by using ELSA (30). CD11b^+^ monocyte/myeloid cell population was sampled for further analysis using Seurat package on Bioconductor (R) (31). Following Elbow Plot analysis, the number of principal components analysis (PCA) was set up to 3 with 0.2 resolution for UMAP clustering.

### Cell Lines

GL261 cells were cultured in Dulbecco’s Modified Eagle Medium (DMEM, Gibco) supplemented with 10% fetal bovine serum (FBS, Hyclone), 1x antimycotic-antimycotic solution (Gibco), 1% L-glutamine, ß-mercaptoethanol, 200 µg sodium pyruvate, and 1x NEAA. Cell lines were kept in a 37°C humidified incubator with 5% CO2. Cell number and viability were measured using the trypan exclusion method (0.4% trypan Blue, Gibco).

### Mice

C57BL/6J mice (Stock No. 000664) and B6.Cg-Thy1 a/Cy Tg(TcraTcrb)8Rest/J (PMEL; Stock No. 005023 (32)) were purchased from the Jackson Laboratory and housed in animal facility of the UPMC Children’s Hospital of Pittsburgh. Animals were kept in the facility for at least one week prior to performing any procedures to minimize stress-related symptoms. 5–6-week-old female were used in the experiments. All experiments were conducted following protocols approved by the University of Pittsburgh Institutional Animal Care and Use Committee (IACUC).

### Intracranial Tumor Model and Antibodies Treatment

Mice were anesthetized by mask inhalation of 1.5% vaporized isoflurane throughout the surgical procedure. GL261 cells (100,000 cells in 2 μL DPBS) were stereotactically implanted into the caudate nucleus using the following coordinates relative to bregma: x = +2.5 mm (lateral), y = +1.5 mm (anterior), and z = 2-3.0 mm (inferior) (33). MRI was performed 7 days post tumor cell implantation to confirm tumor presence, and again at day 40 to measure tumor size growth in control-treated animals and αTIGIT & αPD1 dual blockade-treated animals. All mice were randomized prior to their separation into treatment groups. IgG1 (clone MOPC-21), IgG2a (clone RTK2758), αPD1 (clone RMP1-14) and αTIGIT (clone 1G9) antibodies were obtained from Bio-X-Cell Antibodies were dosed at 200 μg per animal and administered *via* intraperitoneal (i.p.) injection, as described previously (34) for both the survival and immunophenotyping studies. Anti-TIGIT and anti-PD1 treatments were given on the same day twice per week starting on day 8, for a total of 7 doses. Mice were euthanized after receiving seven doses of immune-checkpoint inhibitor therapeutic antibodies (αTIGIT/αPD1) to investigate biological endpoint and immune cell phenotype. Mice were monitored for weight loss and morbidity symptoms for survival study. All survival experiments were repeated in triplicate with at 4-6 animals per group.

### Mouse Immune Cell Isolation

For the biological endpoint study, mice were euthanized on day 22 (CO2 asphyxiation followed by cervical dislocation) post-tumor inoculation. Brains were dissected and processed for flow cytometry analysis. Brains were homogenized in Collagenase IV Cocktail (3.2 mg/mL collagenase type IV, 1.0 mg/mL deoxyribonuclease I, 2 mg/mL Soybean Trypsin Inhibitor). Samples were centrifuged for 5 minutes at 1500 rpm, followed by red-blood cell (RBC) lysis using ACK lysing buffer (Lonza). Cell viability was measured using the trypan blue exclusion method. Cells were resuspended in FACS Buffer (DPBS with 1% BSA) and centrifuged for 5 minutes at 300 g, after which the pellet was resuspended in FACS buffer. The cells were then stained with appropriate antibodies and acquired on a BD LSR Fortessa flow cytometer.

### Isolation of TILs From GBM Patients

Patient-derived GBM tissue was dissociated, using Accutase (1:10), to form a single cell suspension (SCS). SCS was centrifuged at 1500rpm for 5 mins. The pellet was resuspended in 5mL of 70% Percoll solution. A Percoll gradient of 5mL of 37 and 5mL of 30% Percoll sequentially, was then overlaid onto the tumor-containing 70% Percoll solution. The tumor gradient solution was centrifuged at 2400 rpm for 20 minutes. Immune cells at the interphase were collected and washed once with PBS. The cells were then stained with appropriate antibodies and acquired on a BD LSR Fortessa flow cytometer.

### Isolation of PBMCs

Peripheral blood samples were collected in preservative-free heparin tubes (10 U/mL) and layered into an equal volume of Ficoll-Hypaque density gradient solution (Amersham Pharmacia Biotech Ltd., Little Chalfont, UK). Samples were then centrifuged at 2250 rpm for 20 minutes. After removal of the top layer (plasma), the mononuclear cells (PBMCs) were collected and washed twice with PBS (Hyclone™, GE Healthcare). Cell viability was determined by trypan blue exclusion and exceeded 95%. The cells were then stained with appropriate antibodies and acquired on a BD LSR Fortessa flow cytometer.

### Generation of Immunosuppressive Myeloid Cells From Bone Marrow (BM)

Immunosuppressive myeloid cells were generated as described previously (35). Briefly, tibia and femur-derived BM cell from C57BL/6j mice were cultured in complete DMEM media supplemented with 10 ng/ml each of GM-CSF and IL-4. On day 3, floating cells were removed, and medium was replaced with 1:1 complete DMEM media to GL261 tumor-derived conditioned media (TCM), supplemented with GM-CSF and IL-4 for 3 additional days prior to use.

### Suppression of T Cell Proliferation Assay

T cell suppression assay was performed as described previously (21, 36). In brief, hGP100-restricted (B6.Cg-Thy1a/Cy TCR-transgenic) CD8^+^ T cells were isolated from PMEL-mice (32) using magnetic bead separation (Miltenyi Biotec) and labeled with Cell-Trace proliferation dye (Invitrogen. Cat. No C34557) according to the manufacture guidelines. Feeder cells (antigen presenting cells) were generated from non-CD8^+^ cell fraction and were treated with 10 μg/ml of mitomycin at 37°C for 1 hour to cease proliferation (37). T cells and feeder cells were co-cultured with BM-derived immunosuppressive myeloid cells in the presence of 100 U/mL hIL-2 (PeproTech), 100 µg/mL hGP10025-33 peptide (antigen), and 10 µg/mL of either IgG2a (RTK2758) – as control, or αPD1 (RPMI14) and αTIGIT (1G9). Cells were collected and analyzed on day 4 by flow cytometry.

### Flow Cytometry

Prior to cell surface staining, samples were stained with cell viability dyes (GhostDye or 7AAD) in PBS for 20 minutes in 4°C and then washed with FACS buffer. For mouse immune cell staining, the cell suspensions were blocked with 1% anti-mouse Fc-receptor (CD16/CD32) in FACS buffer for 20 minutes, then washed and stained with fluorescently labeled anti-mouse antibodies for 45 minutes in FACS buffer at 4°C. TILs (n=5) and PBMCs from 2 matched, 3 unmatched and 3 healthy donor (HD) patient samples (n=8) were washed with PBS and stained with cell-surface antibodies for 30 minutes at 4°C per the manufacture guidelines. After staining, cells were washed with FACS buffer and fixed with fixation buffer (BD Cytofix/Cytoperm buffer). The cells were washed with FACS buffer, resuspended in FACS buffer and analyzed by flow cytometry. The antibody clones were purchased from BioLegend or eBioscience and used for flow cytometry as follows: For mouse cell staining: CD4 (GK1.5), CD8 (53-6.7), CD11b (M1/70), CD45 (30-F11), Gr-1 (RB6-8C5), CD3 (17A2, and 145-2C11), Granzyme B (QA16A02). For human cell staining: CD45 (2D1), CD11b (ICRF44), CD3 (C3e/1308), CD8 (OKT-8), PD-1 (EH12.2H7), PD-L1 (MIH2), CD33 (WM53), CD226 (11A8), TIGIT (A15153G), CD155/PVR (SKII.4). GhostDyes (TONOBO) UV450 and Red-780, and 7AAD were used to stain for cell viability (live/dead) according to the manufacture guidelines. Gating was performed on live CD45^+^ cells to designate all immune cells. All samples were analyzed on a BD LSRFortessa (BD Biosciences). Data were analyzed using BD FACSDiva (BD Biosciences) and FlowJo V10 data analysis software (FlowJo LLC).

### Statistical Analysis and Software

Kaplan-Meier survival curves were generated to determine survival and then compared using the log-rank Mantel Cox test. One-way analysis of variance (ANOVA) with Kruskal Wallis multiple comparisons test was used to compare assays containing more than two groups. Statistical significance was considered as p <0.05. Normal distribution was assumed unless specified overwise in the text or figure legend. The analyses were performed using GraphPad Prism 8 or Bioconductor (R programing) on RStudio.

## Results

### High Expression Level of Immune Checkpoint Molecules Associated With Overall Survival (OS) and Disease-Free Survival (DFS) in GBM

**Figure 1:**
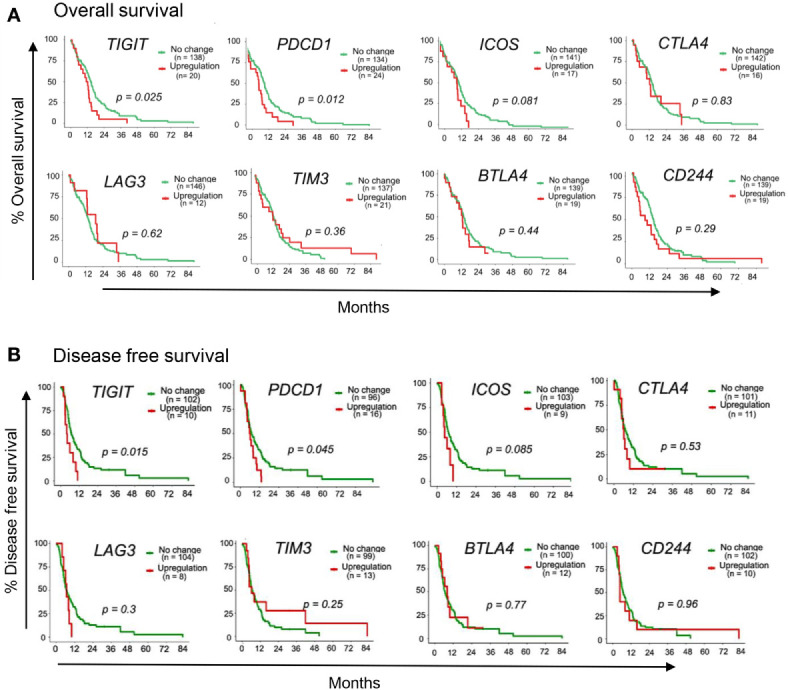
Immune-checkpoint receptor genes associated with GBM patient outcome. TCGA patient survival data obtained from cBioPortal, and patients were grouped based on gene expression z-scores to *upregulated* expression (z ≥2; red line) or *no change* expression (z <2; green line). The **(A)** overall survival rate and **(B)** disease free survival rate, were plotted using Kaplan-Meier survival curves. P values reflect one-way ANOVA with Kruskal Wallis comparison test. n=153.

**Figure 2:**
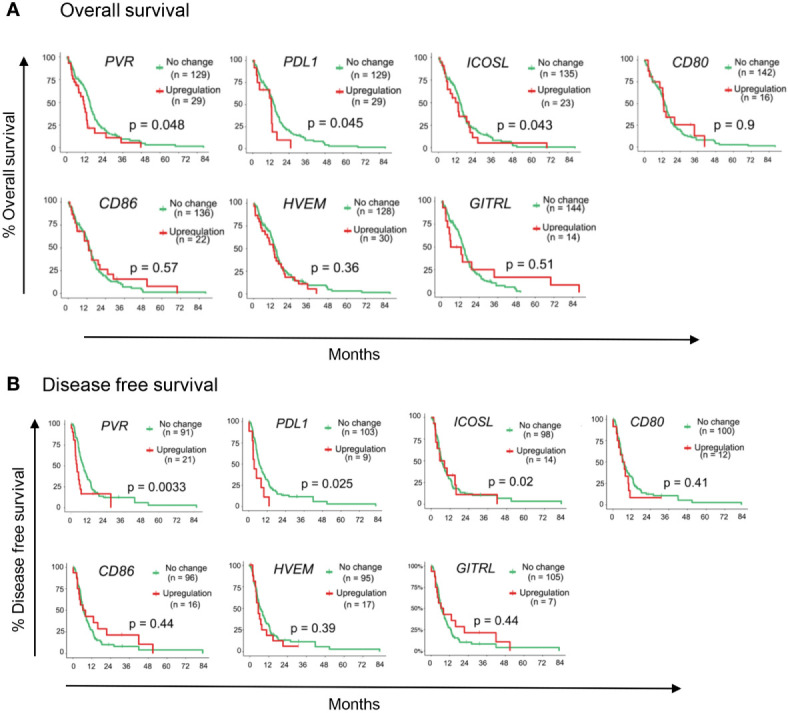
Immune-checkpoint ligand genes associated with GBM patient outcome. TCGA patient survival data obtained from cBioPortal, and patients were grouped based on gene expression z-scores to *upregulated* expression (z ≥2; red line) or *no change* expression (z <2; green line). The **(A)** overall survival rate, and **(B)** disease free survival rates were plotted using Kaplan-Meier survival curves. P values reflect one-way ANOVA with Kruskal Wallis comparison test. n=153.

To identify putative immunotherapy targets for GBM, we evaluated the expression of IC genes and their ligands in RNA sequencing (RNA-seq) data of 153 GBM tumor samples in the TCGA database (38). We first assessed the correlation of 15 established IC gene expression levels with overall survival (OS) and disease-free survival (DFS) (39, 40). Upregulated expression was defined as expression z score greater than 2. Our data demonstrate that upregulated expression (red lines) of *TIGIT* and *PDCD1* (gene encoding PD1) were associated with poor patient outcome and increased mortality as compared with patients who had no change in *TIGIT* and *PDCD1* RNA expression (green lines) (**Figures 1A, B**). Upregulated *ICOS* expression was also associated with reduced OS and DFS, although the data did not reach significance (**Figures 1A, B**). However, upregulated expression of other IC receptor genes, including *CTLA4*, *LAG3*, TIM3 (*HVAC1*), *BTLA4*, and *CD224* were not associated with changes in OS and DFS. Interestingly, expression of genes for *CD155* (PVR), *PD-L1*, and *ICOS-L*, the ligands for TIGIT, PD1, and ICOS, respectively, was also significantly associated with decreased OS and DFS, whereas upregulated expression of other IC gene ligands did not affect these parameters in GBM patients (**Figures 2A, B**). Although our survival analysis assessed patients with elevated expressed based on Z score (i.e. compared to mean expression of that gene), we further examined the absolute expression of each gene to determine the extent of therapeutic utility among all patients. Our data show that a large portion of patients showed to have physiologically relevant expression levels (TPM>1) of the genes encoding to the checkpoint receptors PD-L1 (94%) and CD155 (PVR; 100%) (**Supplemental Figure 1**). Additionally, PD1 and TIGIT were reported to be expressed by large frequencies of GBM CD4^+^ and CD8^+^ TILs (34, 41). Taken together these data suggest that PD1/TIGIT-targeted therapy may be relevant for many patients with GBM.

### TIGIT and PD1 Are Co-Expressed, Share Common Gene Networks, but Are Also Associated With Distinct Pathways in GBM

**Figure 3:**
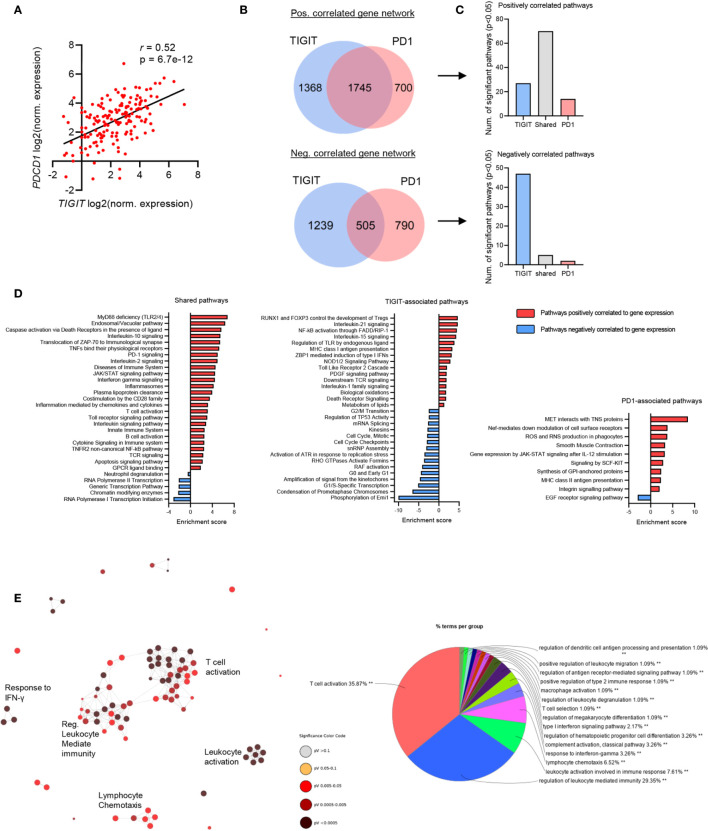
*TIGIT* and *PDCD1* (PD1) exhibit shared immunological networks but have unique regulatory pathways in GBM. GBM patients’ RNA-seq data was obtained from TCGA, transcript per million (TPM) normalized reads were calculated per each patient and Pearson’s correlation analysis was performed. n=153. Genes with a statistically significant (p<0.05 and FDR<0.05) positive correlation and negative correlation to *TIGIT* and *PDCD1* expression were identified. **(A)** Pearson’s correlation analysis of *TIGIT* and *PDCD1* expression. **(B)** Venn diagrams showing number of statistically significant correlated genes unique and overlapping within TIGIT and PD1 gene groups. **(C)** Number of statistically significant (p<0.05 and FDR<0.05) pathway enriched in each corresponding gene group. **(D)** Representative pathways which are positively and negatively enriched in the shared-gene group, TIGIT-associated group, and PD1-associated group. **(E)** Network analysis for Gene Ontology (GO) Immunological Processes associated with *TIGIT* and *PDCD1* positively correlated gene network. Statistically significant gene correlation and pathway enrichments were corrected for false discovery rate (FDR) using Benjamini-Hochberg test.

Our data revealed that TIGIT/CD155 and PD1/PD-L1 checkpoint genes were significantly associated with GBM clinical outcome, thus we next analyzed RNA-seq data from these patients to identify genes and pathways which may be involved with TIGIT and PD1 expression in GBM. Notably, the expression of *TIGIT* and *PDCD1* were significantly correlated with each other (**Figure 3A**), suggesting a rationale for dual blockade of these checkpoint molecules in GBM patients. Despite their significant correlation, *TIGIT* and *PDCD1* may be associated with unique gene networks and pathways (42). Therefore, we next interrogated the gene networks associated with the expression of *TIGIT* and/or *PDCD1* in GBM. We identified a total of 6347 genes which correlated with *TIGIT* and *PDCD1* expression with high statistical significance (p<0.05 and FDR<0.05) (**Figure 3B**). While many genes correlated with both *TIGIT* and *PD1* expression, we also identified a large number of genes and pathways uniquely correlated with either *TIGIT* or *PD1* (**Figures 3B, C** **)**.

TIGIT/PD1 (shared)-associated pathways included immune related pathways, such as Toll-like receptor (TLR) signaling, interleukins signaling (such as IL-10 and IL-2), TCR signaling and T cell activation, and innate immune system pathways (**Figure 3D**). Interestingly, TIGIT-associated pathways included Treg development, MHC class I presentation, caspases and death-receptors signaling, control of cell cycle transition, regulation of TLR and Nf-kB signaling, and p53 regulation (**Figure 3D**). PD1-associated pathways included cell motility, oxidation and phagocytosis, IL-12 mediated Jak-STAT signaling, MHC class II and antigen presentation, and EGF receptor signaling (**Figure 3D**). Immunological network analysis showed that many immune responses were strongly associated with the expression of TIGIT and PD1, mostly T cell activating and regulation of immunity, but also innate immune functions such as leukocytes degranulation, and functions of macrophages and dendritic cells (**Figure 3E**).

Together, these data suggest that upregulated expression of TIGIT and PD1 may confer immunosuppression and tumor aggression in GBM patients through both shared and distinct pathways, and therefore targeting both these pathways may be beneficial for improving clinical outcome of GBM patients.

### Combination of αTIGIT and αPD1 Immunotherapy Increases Numbers of TIL Cytolytic CD8^+^ T Cells and Prolongs Long-Term Survival of GBM-Bearing Mice

**Figure 4:**
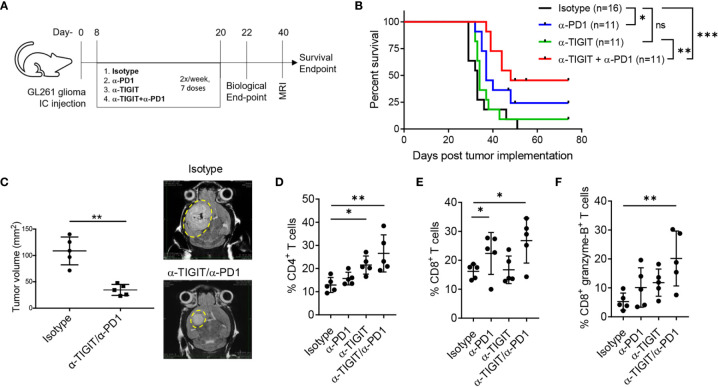
Anti-TIGIT and anti-PD1 combination improves survival of GL261 glioma bearing mice. GL261 glioma cells were injected stereotactically in the caudate putamen of C56BL/6J mice followed by immunotherapy treatment starting on day 8 post tumor injection. Mice were evaluated for T cell responses on day 22 (biological endpoint) and for tumor size by day MRI on day 40. **(A)** Schematic showing induction of GL261 glioma in mice following treatment regimen using anti-PD1 and anti-TIGIT immunotherapies. **(B)** Survival curves with Log-rank (Mantel-Cox) curve comparison test. Pooled data from 3 independent experiments. **(C)** Pooled data and representative MRI images of tumor growth in murine GL261 glioma model in anti-PD1/anti-TIGIT treated group and isotype (control) treated animals. Unpaired *t* test with Welch’s correction. n=5 per group. **(D–F)** Percentages (%) of CD45^+^ glioma-infiltrating CD4^+^ T cells **(D)**, CD8^+^ T cells **(E)**, and CD8^+^ granzyme B^+^ T cells **(F)**, on day 22 following anti-TIGIT/anti-PD1 immunotherapy. Representative data of 3 experiments. n=5 per group. One-way ANOVA with multiple comparisons test corrected for false discovery rate (FDR) using Benjamini-Hochberg test. *P* values are as followed: *≤0.05, **≤0.01, ***≤0.001. NS, not significant.

Our data suggest a beneficial outcome for IC blockade of TIGIT and/or PD1 in GBM. To investigate this hypothesis, C57BL/6 mice were intracranially injected with syngeneic GL261 cells, followed by 7 doses of immunotherapy with (1) isotype control antibodies, (2) αPD1, (3) αTIGIT, or (4) a combination of αTIGIT/αPD1 therapeutic antibodies, administered twice per-week starting on day 8 post-tumor injection (**Figure 4A**). Analysis of immune cells was performed uniformly across groups on day 22 post-tumor implantation (biological endpoint) followed by MRI analysis for tumor size on day 40 for control and dual αTIGIT/αPD1-treatment groups (**Figure 4A**). Control mice (Isotype; black line) displayed median survival of 33 days (range: 29-51 days) with severe morbidity signs and did not reach long-term survival endpoint (**Figure 4B**). While αTIGIT monotherapy (green line) moderately improved survival, treatment with αPD1 (blue line) or a combination treatment of αTIGIT/αPD1 (red line) significantly prolonged animal survival as compared with isotype treated animals (**Figure 4B**). The median survival of αTIGIT treatment was 34 days (range: 32-43 days) while αPD1 monotherapy (green line) was 37 days (range 32-74 days). Notably, αTIGIT/αPD1 dual treatment most significantly prolonged mice survival with median survival of 48 days (range 39-74 days) (**Figure 4B**). MRI analysis showed that in αTIGIT/αPD1 treated animals the tumor size was significantly smaller than tumors in isotype-treated animals (**Figure 4C**). These data confirm previous results in which immunotherapy combination of αPD1 with αTIGIT reduced tumor burden and improved survival of mice with glioma (34).

To examine a mechanism by which the combination therapy improved anti-tumor immunity, we explored the effect of treatment on tumor-infiltrating lymphocytes (TILs) and their cytolytic phenotype on day 22 post tumor implantation. Although αPD1 monotherapy did not significantly affected the percentages of CD4^+^ TILs, treatment with either αTIGIT or αTIGIT/αPD1 resulted in a significant increase of CD4^+^ TILs as compared with control animals (**Figure 4D**). Additionally, we noted a significant increase in percentages of CD8^+^ TILs in tumors in animals treated with αPD1 or αTIGIT/αPD1, but not in αTIGIT-monotherapy treated animals (**Figure 4E**). Analysis showed that treatment with either αPD1 or αTIGIT monotherapy resulted in a mild increase in the percentages of CD8^+^ granzyme-B^+^ TILs, while this effect was significantly increased in αTIGIT/αPD1 combination treatment (**Figure 4F**). These data are complementary to previous results showing that αPD1 or αTIGIT immunotherapy enhances the expression of TNFα and IFNγ in TILs from GBM (34) as well as other cancers (43), and suggest that the therapeutic effect of checkpoint blockade with αTIGIT/αPD1 may work through distinct mechanisms to affect CD4^+^ and CD8^+^ TILs and promote anti-glioma immunity.

### Dual Blockade of TIGIT and PD1 Regulates MDSCs in GBM Murine Model

**Figure 5:**
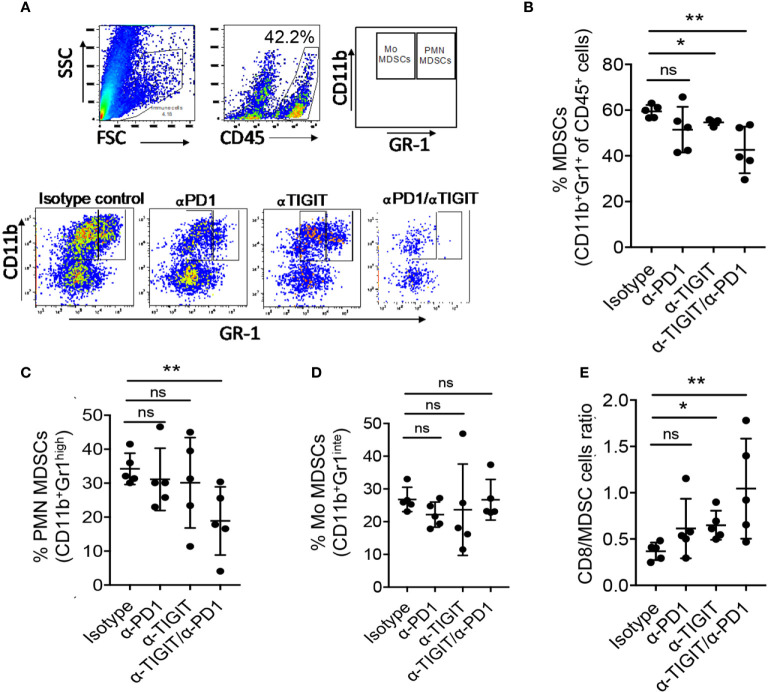
Anti-PD1/TIGIT immunotherapy is associated with altered myeloid-derived suppressor cells (MDSCs) in GL261 murine model. GL261 glioma cells were injected stereotactically in the caudate putamen of C56BL/6J mice followed by immunotherapy treatment starting on day 8 post tumor injection, and the frequencies of MDSCs were determined on day 22. **(A)** Representative flow cytometry plots showing gating strategy of PMN MDCSs and Mo MDCSs based on the expression of Gr1 and CD11b. **(B–D)** Percentages (%) of CD45^+^ glioma-infiltrating total MDSCs (CD11b^+^ Gr1^+^) **(B)**, PMN MDSCs (CD11b^+^ Gr1^high^) **(C)**, and Mo MDSCs (CD11b^+^ Gr1^low^) **(D)**, on day 22 following treatment. **(E)** Ratios of tumor infiltrating CD8+ T cell to total MDSCs. Representative data of 3 experiments. n=5 per group. One-way ANOVA with multiple comparisons test corrected for false discovery rate (FDR) using Benjamini-Hochberg test. *P* values are as followed: *≤0.05, **≤0.01, NS, not significant.

Previous reports have shown that MDSCs stimulate suppressive mechanism to develop a pre-metastatic niche, promote tumor growth, inhibit anti-tumor function of TILs, and negate immunotherapy which results as resistance to IC blockade (44, 45). Furthermore, MDSCs have been shown to contribute to immunosuppressive microenvironment in gliomas, including GBM (45–47). MDSCs were shown to express PD-L1 (48). Additionally, inhibition of TIGIT was reported to abrogate MDSC immunosuppressive capacity *in vitro* (49). Together, these data suggest that targeting PD1 and TIGIT pathways may affect MDSCs in GBM. However, the effects of these checkpoint on MDSC infiltration in gliomas are ill defined (45). We, therefore, investigated if MDSCs were affected by immunotherapy in our model on day 22 (biological endpoint; end of immunotherapy). Shown in **Figure 5A**, glioma infiltrating MDSC subsets were characterized by the expression of Gr1 and CD11b as follows: PMN MDSCs were defined as CD11b^+^Gr1^high^ cells, whereas Mo MDSCs were defined as CD11b^+^Gr1^intermediate (int)^ cells (18). We evaluated the levels of MDSCs and their subsets following immunotherapy (**Figure 5A**; lower panel). Our data show, that compared with isotype treatment, both αTIGIT monotherapy and dual blockade of TIGIT & PD1 significantly reduced the frequencies of GL261 glioma infiltrating MDSCs (CD45^+^CD11b^+^Gr1^+^ cells), most strikingly for αTIGIT/αPD1 combination therapy (**Figure 5B**). Treatment with αPD1 showed a trend of decreasing MDSC percentages, though the results did not achieve statistical significance (**Figure 5B**). Analysis of MDSC subsets revealed that αTIGIT/αPD1 dual treatment significantly decreased the frequencies of PMN MDSCs (**Figure 5C**), while Mo MDSCs levels remained mostly unaltered (**Figure 5D**). Furthermore, we observed a statistically significant increase in ratios of CD8^+^ T cells over total MDSCs in tumors when mice were treated with αTIGIT monotherapy or αTIGIT/αPD1 combination therapy (**Figure 5E**). Blockade of PD1 alone did not significantly increase the CD8^+^ T cells/MDSCs ratios (**Figure 5E**). Together, our data reveal a mechanism of TIGIT/PD1 blockade in glioma and suggest distinct roles of these ICs on MDSC subsets and in regulating tumor immunity.

### Myeloid Cells Upregulate PD-L1 and TIGIT-Ligands in GBM Which Inhibit T Cell Functions

**Figure 6:**
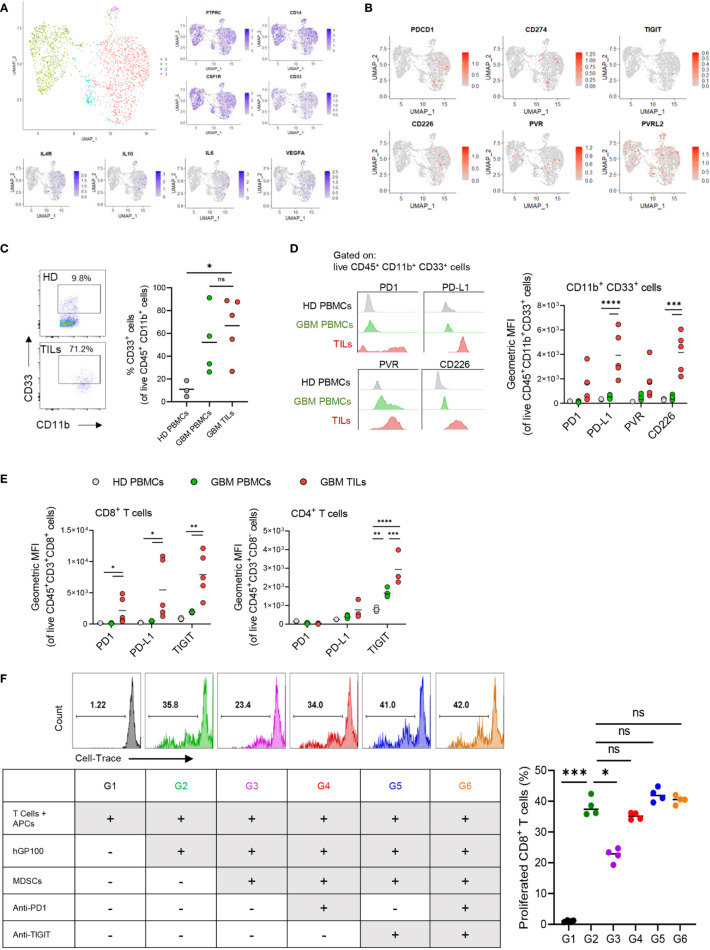
PD1, PD-L1 and TIGIT-ligands are expressed on myeloid suppressor cells in GBM and contribute to T cell dysfunction. Single cell (sc) RNA-seq analysis was performed on myeloid cells from GBM patients. **(A)** UMAP clustering and expression (z-scores) of suppressive myeloid cell markers. **(B)** Expression z-scores of PD1/TIGIT-associated checkpoint molecules in the scRNA-seq clusters. **(C–E)** Healthy donor (HD) PBMCs and GBM patient PBMCs and TILs analyzed flow cytometry for myeloid cells, T cells, and IC markers. n = 4 HD; n = 5 GBM patients. **(C)** Representative flow cytometry plots and percentages (%) of CD11b^+^ CD33^+^ myeloid cells. **(D)** Representative histograms and mean fluorescence intensity (MFI) of PD1, PD-L1, PVR, and CD226 on CD11b^+^ CD33^+^ cells. **(E)** MFI of PD1, PD-L1, PVR, and CD226 on CD8^+^ T cells and CD4^+^ T cells. **(F)** T cell proliferation assay of murine hGP100-reactive CD8^+^ T cells cultured with immunosuppressive myeloid cells with αTIGIT and αPD1. Representative histogram plots and percentages (%) of proliferated CD8^+^ T cells at different culture conditions as indicated in the table lagend. n=4 per group. One-way ANOVA with Tukey multiple comparisons correction. ns, not significant. p = *<0.05, **<0.01, ***<0.001, ****<0.0001.

We next evaluated the potential of αTIGIT/PD1-immunotheraphy to impact MDSC-like cell in GBM patients. For that, we first analyzed single-cell (sc)RNA-seq data of CD11b^+^ myeloid cells from GBM patients (28) for the expression of PD1, TIGIT, and their ligands. Myeloid cells were confirmed based on the expression of CD45 (*PTPRC*), *CD14*, and *CSF1R* genes (**Figure 6A**) (28, 50). Of note, we identified 4 unique clusters of tumor-associated myeloid/macrophage cells (TAMs) in GBM, which had distinct expression profiles (**Figure 6A** and **Supplemental Figure 2A**). The expression of CD33, an hematopoietic progenitor cell marker which commonly used to identify pan-MDSCs (51), was distributed throughout the TAM clusters. Nonetheless, the expression of inhibitory and suppressive markers, including genes for IL-4R, IL-10, IL-6, VEGFA, CCL2 and IL-1β (**Figure 6A** and **Supplemental Figure 2A**), were mostly expressed by cluster 0, suggesting that these cells had a tumor-promoting and immune-suppressing functions, which resemble MDSC-like cells (52). Interestingly, *PDCD1* (PD1), *CD274* (PD-L1), and *CD226*, were also predominantly expressed by cluster 0 (**Figure 6B** and **Supplemental Figure 2B**). CD155 (PVR) was also associated and expressed by cluster 0, although less frequent than CD226, PD1, and PD-L1 **(** **Figure 6B** and **Supplemental Figure 2B**). PVRL2, another inhibitory receptor that bind to TIGIT (53), was also expressed by TAMs, with highest expression levels in cluster 0 (**Figure 6B** and **Supplemental Figure 2B**). We did not detect TIGIT expression in TAMs by scRNA-seq (**Figure 6B**). Additionally, we noted high expression and associated of ICOS-L with cluster 0 (**Supplemental Figure 2B**), which interestingly was also associated with GBM patient OS and DFS (**Figures 2A, B**). These data suggest that immunosuppressive TAMs, such as MDSCs, express genes for PD1, PD-L1 and TIGIT-ligands. Therefore, evaluated the protein expression of these markers on CD45^+^ immune cells in patient derived GBM tissue and PBMCs, and healthy donor (HD) PBMCs. The frequencies of CD11b^+^ CD33^+^ cells in GBM TILs were on average higher compared to GBM PBMCs, and were significantly higher than of HD PBMCs (**Figure 6C** and **Supplemental Figure 2C**), suggesting that MDSCs are present at high levels in GBM and could contribute to the TME immunosuppression (46). Consistent with our scRNA-seq data, CD11b^+^ CD33^+^ cells had higher expression levels of PD1, PD-L1, PVR, and CD226 in TILs, as compared with CD11b^+^ CD33^+^ cells from PBMCs of GBM patients and HDs, most notably for PD-L1 and CD226 expression (**Figure 6D**). Moreover, as compared to PBMCs samples we noted an increased expression of PD1, PD-L1 and TIGIT on CD8^+^ TILs, while CD4^+^ TILs had mostly upregulated expression of TIGIT (**Figure 6E**). Together, these data suggest that CD11b^+^ CD33^+^ TAMs may promote immunosuppressive functions at-least in part through expression of PD1/TIGIT-checkpoint ligands. To test this hypothesis, hGP100-restricted naïve T cells isolated from pmel mice (32) were activated *in vitro* with hGP100_25–33_ peptide and feeder cell (antigen presenting cells; APCs) in the presence of bone-marrow derived myeloid cells (putatively MDSC-like cells) cultured in GL261 cell-derived tumor-conditioned media and treated with αTIGIT and αPD1. Our data indicated that glioma-conditioned immunosuppressive myeloid cells significantly inhibited CD8^+^ T cell proliferation, which was restored by the addition of αTIGIT or αPD1 (**Figure 6F**). In summary, these new data suggest that immunosuppressive myeloid cells, and presumably MDSCs, suppress anti-tumor immunity by inhibiting antigen-specific T cell function in GBM, at least in part *via* TIGIT and PD1 pathways, which may have major implication to patient treatments by immunotherapy.

## Discussion

In cancer, the dysregulation of immune checkpoints, such as TIGIT and PD1, is directly associated with tumor progression and enhanced immune evasion (54–57). In the past decade, an increasing number of IC-targeted immunotherapies have proven to have substantially beneficial outcomes for a wide variety of malignancies and provide durable tumor immunity and long-term patient survival (58). Nonetheless, evidence supporting the efficacy of IC immunotherapy in glioma remains insufficient (11).

In this study we interrogated RNA-seq data of 153 GBM patients in the TCGA database to identify IC genes whose upregulated expression is associated with poor outcome. We found that upregulated expression of *TIGIT* and *PDCD1*, as well as their ligands *CD155* (PVR) and *PD-L1* (respectively), was significantly correlated with poor DFS and OS. Other checkpoint pathways with inhibitors currently in development, including LAG3 and TIM3, were not associated with either patient OS or DFS. We posit that interrogation of TIGIT and PD1 -associated regulatory gene networks in responding and non-responding GBM patients would be of great interest to identify biomarker of ICIs.

PD1 is an immune checkpoint expressed on activated immune cells, including CD4^+^ and CD8^+^ TILs. The binding of PD1 to its ligand, PD-L1 on tumor and stromal cells, delivers a signal that inhibits effector functions such as cytokine production and cytolytic activity in the tumor microenvironment (TME) (59). PD-L1, like many other IC ligands, is hijacked by tumor cells in order to evade anti-tumor immunity. Accordingly, blockade of PD1/PD-L1 pathway with antibodies have been shown to improve T cell function and reduce tumor burden in several types of tumors (60, 61). Previous studies demonstrated elevated levels of TIGIT expression in human gliomas (34); however, the therapeutic effects of targeting this pathway in glioma patients remain poorly understood. TIGIT has recently emerged as an important checkpoint that is also expressed by activated CD4^+^ and CD8^+^ TILs. TIGIT has a higher binding affinity to CD155 than CD226; thus, once TIGIT is upregulated, the inhibitory signal becomes more dominant (62–64). Similarly, interfering with TIGIT/CD155 interaction has been identified as a potential therapeutic target for malignancies (65). Interestingly, blocking PD1/PD-L1 signaling was shown to increase the expression of TIGIT on Tregs in head and neck squamous cell carcinoma (HNSCC) patients (49), suggesting a resistant mechanism for αPD1 immunotherapy mediated by TIGIT.

Accordingly, our data support prior studies that combination immunotherapy treatment targeting the PD1 and TIGIT pathways leads to prolonged survival in GBM murine models (34, 66). Furthermore, we showed that αTIGIT/αPD1 dual treatment increased the numbers of CD8^+^ TILs and enhanced their lytic function in GBM, supporting previous findings that this treatment can enhances IFN-γ expression in glioma-infiltrating T cells (34). Importantly, our data indicate that combined immunotherapy with αTIGIT/αPD1 affects MDSCs in the glioma TME.

MDSCs are a heterogenous population of immature myeloid cells that contribute to tumor growth, accumulation of additional immunosuppressive cells, and immunotherapy resistance (66, 67). Furthermore, MDSCs express large amounts of immunosuppressive factors, multiple anti-inflammatory cytokines and chemokines that directly stimulate tumor progression (68). Notably, a long-term survival study in melanoma patients showed that elevated numbers of MDSCs were highly associated with ICI resistance and negative therapeutic outcomes (69). Additionally, elevated numbers of tumor infiltrating MDSCs are correlated with CD8^+^ TIL dysfunction and induced tumor cell expression of IC ligands; thus, MDSCs may promote and sustain an immunosuppressive glioma TME (70–72). Here, we showed that TIGIT blockade stimulated anti-tumor cytotoxic T cell (CTL) responses and reduced the immunosuppressive MDSCs in a murine model of GBM. Moreover, we found that PMN MDSC, but not Mo MDSC accumulation was reduced by dual blockade of TIGIT and PD1, compared with controls. Thus, our data suggest that PMN and Mo MDSCs might have different mechanisms to confer resistance against ICI immunotherapy, but may also be a target of ICI in glioma. We posited that future studies should focus on unveiling the crosstalk and mechanisms by which ICIs affect MDSCs in glioma. Along these lines, we showed that suppressive myeloid cells express PD1, PD-L1, and TIGIT-ligands in human GBM tissue. Moreover, we demonstrated that antigen specific T cell proliferation is inhibited by immunosuppressive myeloid cells can be restored by TIGIT/PD1 blockade. This suggests that CTL exhaustion might be regulated at least in part by the expression of IC ligands on MDSCs in GBM.

Treg cells are major components of the immune suppressive TME which express many ICs (73). The expression of TIGIT and PD1 by Treg cells was shown to enhances their immunosuppressive functions and contribute to tumor progression both in glioma murine models and GBM patients (74). Importantly, Treg cells are major source of IL-10 in GBM (74, 75), and IL-10 can induce MDSC development and enhance their suppressive functions (76, 77), as well as increasing the expression of PD1 myeloid cells (78). Additionally, TIGIT is important for IL-10 expression by Treg cells (55). Therefore, it is possible that αTIGIT might also regulate MDSC cell numbers and functions by suppressing Treg expression of IL-10. Future studies should focus on the mechanisms and crosstalk between Treg cells and MDSCs *via* checkpoint molecules in the GBM TME and their contribution to ICI resistant.

In summary, our data support the concept of treating GBM patients with dual blockade of PD1 and TIGIT and provides new insights into mechanisms of GBM immunotherapy to facilitates the development of novel treatments.

## Data Availability Statement

The datasets presented in this study can be found in https://www.ncbi.nlm.nih.gov/gap/ online repository, under accession number phs000178.

## Ethics Statement

The animal study was reviewed and approved by University of Pittsburgh Institutional Animal Care and Use Committee.

## Author Contributions

Performed experiments and collected data: IR, RK, LM, KS, PS, CS, JN, NA, MLC, AF and TB. Formal data analysis and figures: IR and RK. Statistical analysis: IR, SZ, and DR. RNA-seq and single cell RNA-seq analysis: IR, LW, and DR. Resources, concepts and/or manuscript revisions: IR, BH, SA, AB, FSL, IP, NA, BC, AD and GK. IR wrote the manuscript. IR revised the manuscript with assistance from GK. IR and GK designed the experiments. GK supervised and financed the study. All authors contributed to the article and approved the submitted version.

## Funding

This research was supported by National Institute of Health (NIH)/National Cancer Institute (NCI) grants R01CA244520 and NIH R01CA222804 (to GK), NIH/National Institute of Biomedical Imaging and Bioengineering (NIBIB) R21EB029650 (to GK), The Walter L.Copeland Fund of The Pittsburgh Foundation (to IR, GK) and The Brain Tumor Funders’ Collaborative (GK). IR was supported by a fellowship from UPMC Children’s Hospital of Pittsburgh. This work utilized the Hillman Cancer Center Flow Cytometry Core, a shared resource at the University of Pittsburgh supported by the CCSG P30 CA047904.

## Conflict of Interest

The authors declare that the research was conducted in the absence of any commercial or financial relationships that could be construed as a potential conflict of interest.
