# Supplementary material for: TIGIT and PD-1 Immune Checkpoint Pathways Are Associated With Patient Outcome and Anti-Tumor Immunity in Glioblastoma
Source: Front Immunol. 2021 May 7;12:637146. doi: 10.3389/fimmu.2021.637146 (PMC8137816; doi:10.3389/fimmu.2021.637146)
Supplement: Supplementary file 1 [file Presentation_1.pdf]

Supplementary Figure. 1

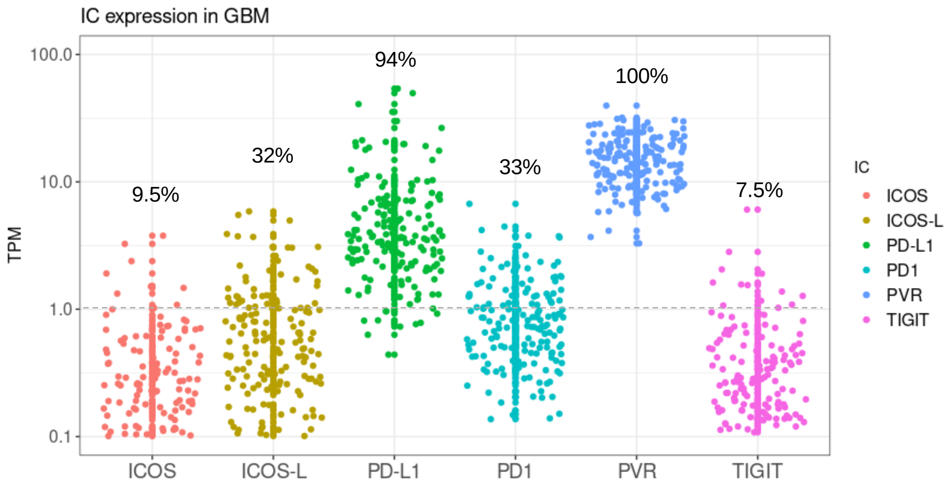

Expression of Immune-checkpoint (IC) genes in GBM. 153 GBM patients' RNA-seq data was obtained from TCGA and transcript per million (TPM) normalized reads were calculated. Shown are scatter plot of TPM expression values for *ICOS*, *ICOS-L*, *PD-L1*, *PD1*, *PVR* (CD155), and *TIGIT*. Percentages (%) reflect number of individuals with gene expression levels greater than 1 TPM (dashed line).

Supplementary Figure. 2

A.

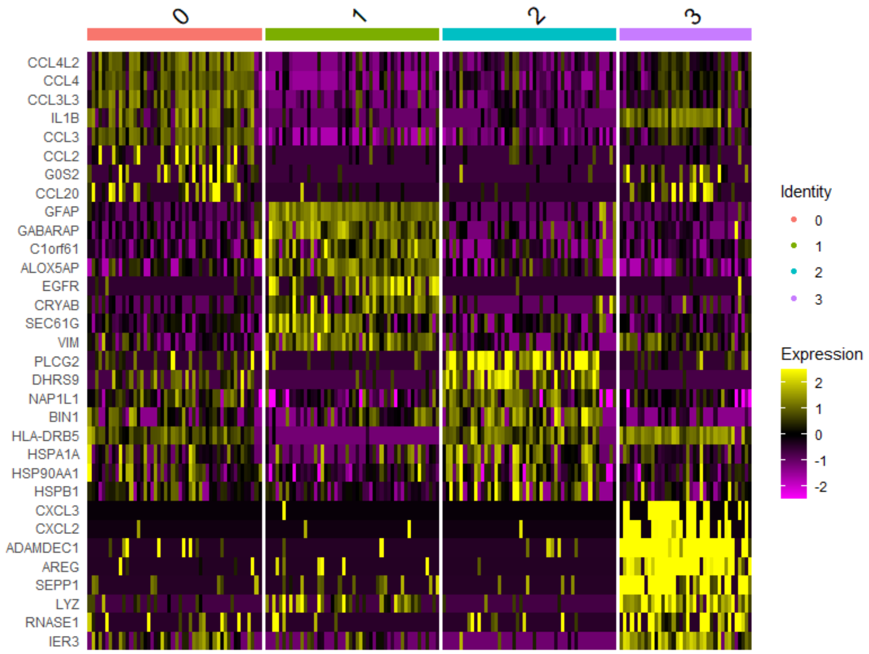

B.

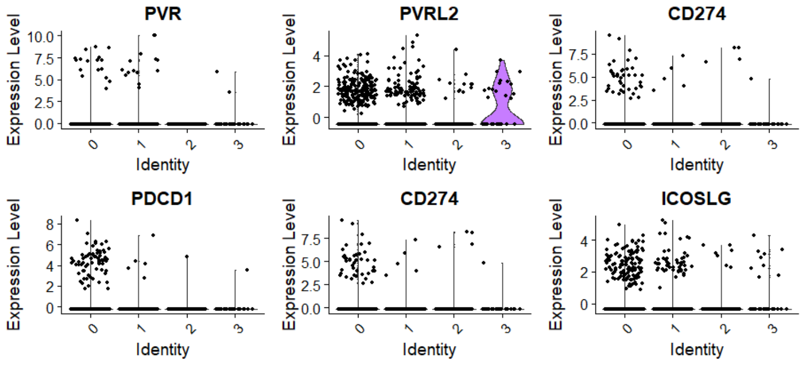

C.

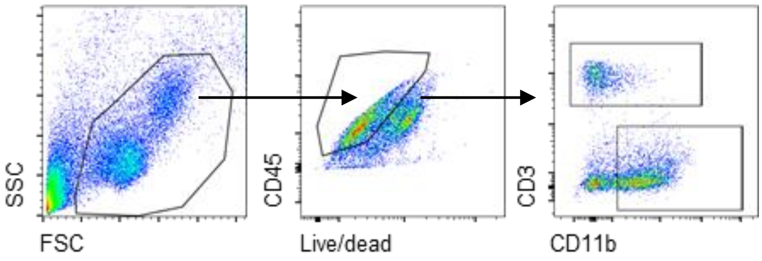

Immunosuppressor myeloid cell analysis in GBM. UMAP clustering analysis (PCA=3) was performed on single cell (sc) RNA-seq of myeloid cells from GBM patients. (A) Z-score expression heatmap of top 8 unique genes identified in each one of the clusters. (B) Expression levels (reads) of PD1/TIGIT-associated checkpoint molecules in the scRNA-seq clusters. (C) Representative flow dot-plot gating strategy of immune cells from PBMC and TIL cells Gating as follow: lymphocytes (SSC vs. FSC), live immune-cells (live/dead Ghost-Dye vs. CD45), T cell and myeloid cells (CD3 vs. CD11b).
